# An outbreak of multi-drug resistant *Escherichia coli* urinary tract infection in an elderly population: a case-control study of risk factors

**DOI:** 10.1186/s12879-015-0974-0

**Published:** 2015-06-09

**Authors:** Rosemary Ikram, Rebecca Psutka, Alison Carter, Patricia Priest

**Affiliations:** Microbiology, Christchurch School of Medicine University of Otago, Christchurch, New Zealand; Department of Preventive and Social Medicine, University of Otago, Dunedin, New Zealand

**Keywords:** Drug resistance, Bacterial, Hospital, Nursing home, Risk factors, Antimicrobial prescribing, UTI, Escherichia coli

## Abstract

**Background:**

Prevention of infection due to multi-drug resistant organisms is particularly challenging because of the spread of resistant bacteria beyond hospitals into the community, including nursing homes. This study aimed to identify risk factors for the acquisition of a multidrug resistant (MDR) *Escherichia coli* in a local outbreak.

**Methods:**

Study participants were all aged over 65 years. Cases had the MDR *E. coli* isolated from a routine urine sample, and controls had a urine sample submitted to the laboratory in the same time period but the MDR *E. coli* was not isolated. Information from clinical records was used to identify risk factors both in the hospital and the community setting for acquisition of the MDR *E. coli*.

**Results:**

76 cases and 156 controls were identified and included in the study. In a multivariate analysis, risk factors statistically significantly associated with acquisition of the MDR *E. coli* were female gender (adjusted OR 3.2; 95 % confidence interval 1.5–6.9), level of care (high dependency OR 7.5; 2.2–25.7) compared with living independently), and in hospital prescription of antimicrobials to which the MDR *E. coli* was resistant (OR 5.6; 2.5-12.9).

**Conclusions:**

The major risk factors for the acquisition of a MDR *E. coli* were found to be residence in a nursing home and in-hospital prescription of antimicrobials to which the MDR *E. coli* was resistant. This emphasises that prevention of transmission of MDROs within a community needs to involve both hospitals and also other healthcare organizations, in this case nursing homes.

## Background

Urinary tract infections (UTIs) are the most common bacterial infection in older populations [1]. Globally there are an estimated 150 million UTIs each year leading to more than 6 billion dollars in direct healthcare costs [2]. Although for most healthy adults UTIs are an inconvenience requiring short-term antimicrobial therapy, in patients with pyelonephritis including the elderly this can lead to bacteremia, hospitalisation, systemic antimicrobial therapy, decreased functional status and death [3].

Multi-drug resistant organisms (MDRO) add further complications which result in decreased effectiveness of standard treatments [4]. The control of UTIs due to MDROs can be challenging in particular due to the frequent emergence of resistance to new antimicrobials and the spread of resistant bacteria beyond hospitals into the community including nursing homes [5]. These facilities can act as reservoirs of these MDROs which can then be transmitted to acute hospitals [5, 6]. In vulnerable populations, including nursing home residents where inappropriate treatment of asymptomatic bacteriurua may be a particular issue [7], UTIs can have higher prevalence of resistance to antimicrobials including ciprofloxacin, cephazolin, and nitrofurantoin [8]. Mortality is higher following bacteremia with strains resistant to antimicrobials than with strains sensitive to antimicrobials [9], although this is most likely due to inappropriate empiric antimicrobial therapy rather than an association with increased virulence of the *E coli* strains [10, 11].

Risk factors for diagnosis of UTIs caused by MDROs can be classified as individual factors, predisposing factors, and bacterial factors [3]. Individual or demographic characteristics that may affect risk of colonization of the urinary tract with MDROs include older age [8, 12–14], female sex [8], a history of UTIs [13, 15], and diagnoses of dementia or poor functional level [5, 14], diabetes [12, 13], and prostatic disease. Predisposing or healthcare-associated factors associated with increased risk of community acquired MDROs in the urinary tract include invasive procedures such as urinary catheterisation [13, 16], previous hospitalisation [12, 13, 15], residence in a nursing home [8], and prior exposure to antimicrobials [5, 12–19].

From 30 March 2009 to 30 June 2011 a phenotypically distinct MDR *Escherichia coli (E.coli)*, which failed to ferment lactose on MacConkey agar and was resistant to gentamicin, quinolones including fluoroquinolones, trimethoprim, trimethoprim-sulfamethoxazole, and amoxicillin, and in this case susceptible only to nitrofurantoin and cephalosporins, was identified as the cause of 77 UTIs in South Canterbury. This is a semi-rural region in New Zealand, population 58,000. The first diagnosis of this organism was in 2007, but in 2009 the prevalence of fluoroquinolone resistance increased from 5 % *E.coli* isolates in 2007 to a peak of 13 % resistance in 2009 and 2010 decreasing, after interventions to 9 % in 2011. In 2009 this necessitated a change in the antimicrobial formulary to cover this MDR *E. coli* both for bacteraemia, with the urinary tract as the potential source, and also treatment for cystitis *.* Infection prevention and control strategies were introduced with a view to decreasing the transmission of this organism. These were; placing alerts on the medical records and implementing infection prevention and control precautions for cases with MDR *E.coli* with this phenotype in both the acute hospital and nursing homes, educating general practitioners with a view to reducing fluoroquinolone use and education sessions on infection prevention and control procedures for the staff of the local nursing homes.

This study aimed to identify risk factors associated with the acquisition of this MDR *E. coli*, to provide evidence to support infection control and antimicrobial stewardship strategies to reduce future transmission.

## Methods

### Study population and setting

We conducted a case-control study to investigate risk factors for diagnosis of the UTI due to this MDR *E.coli* in the South Canterbury region of New Zealand. All cases and controls lived in this region, were 65 years or older, and had submitted a urine sample for microbiology.

### Laboratory methods

Multidrug resistant E. coli was define as being resistant to at least 3 classes of antimicrobial agent. In this case the MDR *E. coli* was isolated by routine laboratory culture by plating 0.001 ml urine onto 5 % sheep blood agar (BD Difco Fort Richard Auckland) and MacConkey agar (BD Difco Fort Richard Auckland). This organism was phenotypically distinct because it failed to ferment lactose and was resistant to amoxicillin, trimethoprim, norfloxacin, genatamicin and with variable susceptibility to amoxicillin clavulanate. It remained susceptible to nitrofurantoin and cephalosporins. It was not an extended β lactamase producer. Susceptibility to antimicrobials was performed using the Clinical Laboratory Standard Institute (CLSI) disc method.

#### Molecular methods

Pulsed-field gel electrophoresis of *Xba*I- digested genomic DNA was performed, at the ESR Porirua, as previously described [20]. PFGE banding patterns were analysed using BioNumerics software version 6.6 (Applied Maths, St-Martens-Latem, Belgium), with the Dice coefficient and unweighted- pair group method with arithmetric averages, at settings of 0.5 % optimization and 1.5 % position tolerance. Multilocus sequence typing (MLST) was performed on isolates from 5 patients with phenotypically characteristic features [21], sequence types (ST) assigned, as described at http://mlst.warwick.ac.uk/mlst/dbs/dbs/Ecoli. Sequences were analysed using BioNumerics software version 6.6.

### Selection of cases

The cases were all individuals in this region meeting the age criteria with a urine sample from which the MDR *E. coli* was isolated between March 2009 and June 2011. Cases were selected retrospectively using urine results.

### Selection of controls

Controls were chosen from the laboratory work sheet. For each case 2 controls were selected, being the samples from patients aged over 65, where the MDR *E. coli* was not isolated, before and after the case sample on the work sheet list. This was not intended to match controls to cases – it was a pragmatic way of obtaining a representative sample of potential controls in a situation where it was not possible to enumerate all eligible controls and randomly select from the list. Those selected as controls included those where another organism was isolated as well as those where culture was negative. All urines are from patients with suspected urinary tract infection, or suspected colonisation preoperatively.

### Data collection

Structured data forms were used to record data from laboratory microbiology, hospital admission records, and from each individual’s General Practitioner (GP). The following data were collected: demographic data, whether the person lived in their own home or a residential facility, the level of care received, and hospitalizations, antimicrobial prescriptions, and UTI diagnoses in the previous 12 months. Antimicrobial prescriptions were identified as being during a hospitalization (‘hospital prescriptions’) or not during a hospitalization (‘GP prescriptions’ – this included prescriptions by emergency and outpatient departments, where no hospital admission ensued).

### Statistical methods

Data were analysed using Stata Statistic 12 [22] Version Release 12. College Station, TX: StataCorp LP; 2011. and two-sided *p*-values of <0.05 were considered statistically significant. Categorical variables were compared using Chi-squared tests.

Logistic regression was used to assess the effect of risk factors on MDR *E.coli* infection. All variables with a *p* < 0.2 for the univariate relationship with the outcome were considered for inclusion in the logistic regression model. ‘Level of care’ (independent, assisted care, high dependency care) was used as the measure of physical frailty. Where level of care was missing, if the person lived in their own home they were categorized as ‘independent’, and if they lived in a residential facility they were categorized as ‘assisted living’. ‘Recurrent UTI’ was defined as three or more UTIs in the past 12 months. Antibiotic use was assessed both as a ‘yes/no’ variable, and as number of courses in the 12 months prior to the date of submission of the urine specimen. We included prescription only of antimicrobials to which the MDR *E coli* was resistant, since antimicrobials to which it was sensitive would not be expected to increase the risk of infection.

### Ethical approval

This study was approved by the New Zealand Multi-region Ethics Committee (MEC/12/EXP/007).

## Results

**Fig. 1:**
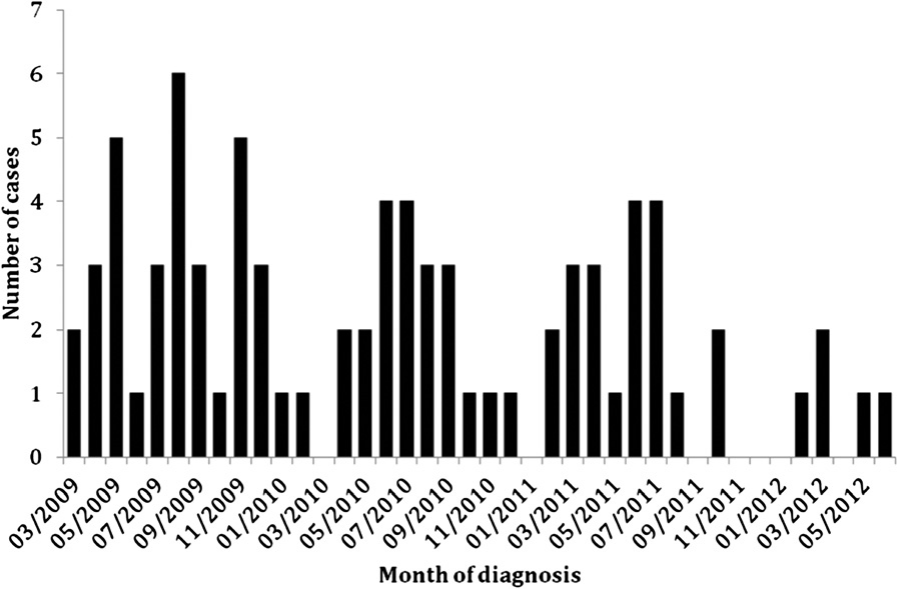
Epidemic curve

Figure 1 shows the epidemic curve for the outbreak. The 5 MLST typed isolates were all E.coli ST 131. We initially identified 78 cases and 156 controls, but on review of hospital notes found that two of the cases were younger than 65 years and therefore ineligible to be cases in this study. Therefore the analysis includes data from 76 cases and 156 controls.

The characteristics of the cases and controls are shown in Table 1. Compared with controls, cases were more likely to be female, be older, live in a residential facility, have been admitted to a hospital in the past 12 months, be diabetic, have been catheterized in the past 12 months, and to have had more UTIs in the past 12 months.

**Table 1:** Characteristics of cases and controls

	Cases	Controls	p*
	(*N* = 76)	(*N* = 156^*^)	
	n (%)	n (%)	
Gender			<0.001
Male	16 (21)	75 (48)	
Female	60 (78)	80 (51)	
Missing	0	1 (1)	
Age			0.005
65–74	14 (18)	52 (33)	
75–84	29 (38)	65 (42)	
85+	33 (43)	37 (24)	
Missing	0	2 (1)	
Residence			<0.001
Own home	22 (29)	109 (70)	
Residential facility	53 (70)	47 (30)	
Missing	1 (1)	0	
Level of care			<0.001
Independent	25 (32)	104 (67)	
Assisted living	38 (50)	46 (30)	
High Dependency	12 (16)	6 (4)	
Missing	1 (1)	0	
Admitted to hospital in past year			0.01
Yes	54 (71)	84 (54)	
No	21 (28)	71 (46)	
Missing	1 (1)	1 (1)	
Diabetes			0.014
Yes	23 (30)	26 (17)	
No diabetes diagnosis	46 (61)	117 (75)	
Missing	7 (9)	13 (8)	
Catheterized in past year			<0.001
Yes	29 (38)	23 (15)	
No	38 (50)	109 (70)	
Missing	9 (12)	24 (15)	
Number of UTIs in past year			<0.001
None	0 (0)	77 (49)	
1	23 (30)	26 (17)	
2	25 (33)	15 (10)	
3+	26 (34)	23 (15)	
Missing	2 (3)	15 (10)	

* *p* value for Chi squared test calculated excluding missing values

Table 2 shows antimicrobial prescriptions from all settings, GP practices, and in hospital for both cases and controls. Compared with controls, cases were overall more likely to have had prescriptions of any antimicrobials and of antimicrobials to which the MDR *E coli* was resistant. This pattern was driven by prescribing in hospitals, and in contrast, in the GP setting about the same proportion of cases and controls had been prescribed any antimicrobials and antimicrobials to which the MDR *E coli* was resistant.

**Table 2:** Antimicrobial prescriptions over past 12 months; cases and controls

	Cases (*N* = 76)	Controls (*N* = 156)	*p*
	n (%)	n (%)	
Prescribed in any setting			
Any antimicrobial prescriptions	68 (89)	111 (71)	<0.001
Any ‘resistant’	57 (75)	80 (51)	0.019
GP prescriptions			
Any antimicrobial prescriptions	44 (58)	85 (54)	0.624
Any ‘resistant’	30 (39)	62 (40)	0.919
Cephalosporins	5 (7)	7 (5)	
Macrolides	6 (8)	11 (7)	
Nitrofurantoin	10 (13)	9 (6)	
Penicillins	17 (22)	51 (33)	
Amoxicillin	9 (12)	20 (13)	
Quinolones	16 (21)	27 (17)	
Tetracyclines	4 (5)	11 (7)	
Trimethoprim^b^	18 (24)	29 (19)	
Hospital prescriptions^c^	*N* = 54	*N* = 84	
Any antimicrobial prescriptions	45 (83)	52 (62)	0.007
Any ‘resistant’	38 (70)	28 (33)	<0.001
Gentamicin	11 (20)	7 (8)	
Cephalosporins	13 (24)	26 (31)	
Macrolides	6 (11)	14 (17)	
Nitrofurantoin	7 (13)	3 (4)	
Penicillins	25 (46)	18 (21)	
Amoxicillin	7 (13)	5 (6)	
Quinolones	26 (48)	18 (21)	
Tetracyclines	3 (6)	1 (1)	
Trimethoprim^b^	12 (22)	8 (10)	

^a^ ‘Resistant’ = antimicrobials to which the MDR *E coli* was resistant, i.e. gentamicin, quinolones, trimethoprim, trimethoprim-sulfamethoxazole, and amoxicillin

^b^ Trimethoprim or trimethoprim-sulfamethoxazole

^c^ Among participants who had been hospitalized

The main logistic regression model included the risk factors hospitalization, hospital prescription of ‘resistant’ antimicrobials, age, gender, diabetes, level of care, and recurrent UTI. A second logistic regression was performed that maximized the number of participants included by excluding diabetes and recurrent UTI, which both had more than 7 % missing values. Neither model included GP antimicrobial prescriptions, as they were not different between cases and controls, or a history of catheterisation, because 14 % of participants had missing data on this variable.

The results of the logistic regression modelling are shown in Tables 3 and 4. The highest odds ratios were for prescription in hospital of antibiotics to which the organism is resistant, and requiring a high level of care in the usual living situation. When the number of prescriptions in hospital for ‘resistant’ antibiotics was included in the model, the risk was highest in those with 3 or more prescriptions. Diabetes, recurrent UTI, and female gender all had similar odds ratios of between 2 and 3, and other ORs did not change markedly when diabetes and recurrent UTI were excluded from the model (allowing the model to include the 34 people who had missing values on one of those). Hospital admission and age were not related to the risk of MDR *E coli* UTI independently of the other risk factors. In a further logistic regression model including only the 177 participants with data on catheterisation, the OR for catheterisation in the past year was 2.45 (95 % CI 1.0–5.9). ORs for other variables were not meaningfully different with catheterisation included or not included in the model (data not shown).

**Table 3:** Adjusted model of risk factors for MDR *E coli* infection, antibiotics as yes/no variable

	OR	95 % CI
Full model (*N* = 192)		
Prescription of resistant^a^ antibiotics in hospital	4.4	1.8–11.2
Level of care		
Independent	1.0	
Assisted living	2.3	1.1–4.9
High dependency	10.2	2.5–42.5
Hospital admission	1.0	0.4–2.5
Recurrent UTI	2.2	1.0–4.9
Diabetes	2.4	1.0–5.7
Female gender	2.7	1.2–6.2
Age		
65–74	1.0	
75–84	1.5	0.6–4.1
85+	2.1	0.7–6.1
Excluding diabetes and recurrent UTI (*N* = 226)		
Prescription of resistant^a^ antibiotics in hospital	5.6	2.5–12.9
Level of care		
Independent	1.0	
Assisted living	2.3	1.1–4.9
High dependency	7.5	2.2–25.7
Hospital admission	1.1	0.5–2.5
Female gender	3.2	1.5–6.9
Age		
65–74	1.0	
75–84	1.4	0.6–3.6
85+	2.3	0.9–6.1

^a^ ‘Resistant’ = antimicrobials to which the MDR *E coli* was resistant, i.e. gentamicin, quinolones, trimethoprim, trimethoprim-sulfamethoxazole, and amoxicillin

**Table 4:** Adjusted model of risk factors for MDR *E coli* infection, antibiotics as number of courses

	OR	95 % CI
Full model (*N* = 192)		
Courses of resistant^a^ antibiotics in hospital		
None	1.0	
1	2.9	1.0–8.3
2	2.6	0.7–9.7
3+	6.5	1.4–30.5
Level of care		
Independent	1.0	
Assisted living	2.6	1.1–6.2
High dependency	9.4	2.3–38.7
Hospital admission	1.2	0.5–2.9
Recurrent UTI	2.1	0.9–4.7
Diabetes	2.6	1.1–6.1
Female gender	2.6	1.1–6.0
Age		
65–74	1.0	
75–84	1.5	0.5–3.9
85+	2.1	0.7–6.1
Excluding diabetes and recurrent UTI (*N* = 226)		
Courses of resistant^a^ antibiotics in hospital		
None	1.0	
1	4.0	1.5–10.5
2	4.4	1.3–15.6
3+	6.5	1.6–25.9
Level of care		
Independent	1.0	
Assisted living	2.1	1.0–4.4
High dependency	6.9	2.0–23.2
Hospital admission	1.3	0.6–2.8
Female gender	3.2	1.5–6.8
Age		
65–74	1.0	
75–84	1.4	0.6–3.5
85+	2.4	0.9–6.3

^a^ ‘Resistant’ = antimicrobials to which the MDR *E coli* was resistant, i.e. gentamicin, quinolones, trimethoprim, trimethoprim-sulfamethoxazole, and amoxicillin

## Discussion

This case control study was performed to identify the local risk factors for acquisition of this MDR *E. coli* with a view to using the identified risk factors to target infection control and antimicrobial stewardship interventions In this small community AMR is rarely encountered however, with the emergence of MDROs worldwide and increasing travel within countries as well as internationally, the introduction of MDROs is an increasing risk to any geographical area [23]. Control of such outbreaks with antimicrobial stewardship and infection prevention and control are important strategies to slow the emergence of MDROs at a local, national and global level. In current healthcare environments this needs to be accomplished with minimal utilization of resources.

*E.coli* ST 131 is most commonly associated with community acquired infection [24], although more recently an association with healthcare and elderly patients has been described [25]. This association is mirrored in the outbreak setting in this study. The antimicrobial susceptibility of *E. coli* has been shown to vary geographically and this may be associated with residence in nursing homes [26].

### Major findings

This case-control study found individual and predisposing risk factors that were associated with the outbreak. Individual risk factors that showed an association with the MDR *E. coli* UTI included being female, having diabetes, having recurrent UTIs and requiring a higher level of care all of which have been described in previous studies [5, 8, 12–15]. The odds ratios associated with female gender, diabetes, recurrent UTIs, and age over 85 were around 2 to 3 in all analyses. Catheterisation increased the risk to a similar degree, but predisposing factors with much higher ORs were high dependency care and prescriptions of antimicrobials to which the MDR *E. coli* was resistant in hospital, particularly 3 or more courses.

### Strengths

#### Selection of controls

In case control studies, controls should be selected from the source population independently of their exposure status. Case control studies of antimicrobial resistant infections that use people infected with sensitive organisms as controls [12, 14–19] give biased measurements of the risk associated with antimicrobial prescription. This is because people who were infected with sensitive organisms but received antimicrobials before specimens were obtained, will no longer have sensitive organisms in their specimen and will not be eligible as controls, so the prevalence of prior antimicrobial use in controls will be artificially low [27]. In this study, both cases and controls come from a community-based population of individuals who required a urine test, so controls should represent the same population that gave rise to the cases.

### Limitations

Typing was performed on 5 isolates to investigate this phenotypically distinct MDR E.coli emerging in a population with otherwise low rates of MDR. They were all the same MLST type; E.coli ST 131, and further typing was not performed in this clinical context. Typing of more than the 5 isolates may have confirmed a clonal outbreak.

Although the sample provided sufficient power to find statistically significant associations of a number of risk factors, the confidence intervals are generally fairly wide. A larger study would be necessary to obtain more precise estimates of the importance of catheterisation, diabetes and recurrent UTI for the risk of this infection, but the additional information would not affect our findings that antimicrobial prescription in hospital, and level of care are the strongest risk factors. Because the control selection process meant that cases and controls’ specimens had been submitted at similar times, we could not assess whether there was an effect of the interventions that were put in place in June 2010, on the risk of infection with the MDR *E. coli*.

### Implications

Most of the risk factors identified in this study are not modifiable as part of a strategy to prevent resistant UTIs. It is not clear why female gender would increase the risk of a resistant UTI in a population of people who all had some reason for providing a urine specimen, unless perhaps the number of men in the control group was inflated by men with prostatism whose doctors wanted to exclude a UTI. The other risk factors are reflective of frailty, poor health, and dependence on others for personal care.

An increased risk of resistant UTI may reflect selection of resistant organisms (through previous antibiotic use), or transmission of resistant organisms from others. We were not able to assess whether the antibiotics prescribed in hospital were all necessary, but this study supports the importance of the prudent use of antimicrobials in minimizing the risk of resistant infection. Care of dependent elderly people in nursing homes may be a factor in transmission, and underlines the importance of infection control measures, including hand hygiene in this setting. Also, although hospitalization *per se* did not have an effect on infection independent of antibiotic prescriptions in hospital, only hospital and not community prescriptions increased the risk of the MDR *E. coli* UTI. This suggests that there may be a component of the hospital environment that is important, perhaps the opportunities for transmission of resistant organisms afforded by the care of dependent elderly people in hospital.

This study indicates the importance of multiple healthcare settings in the transmission of this MDR E. coli. Local antimicrobial stewardship and infection prevention and control strategies to control MDROs need to be coordinated across multiple sectors to obtain optimal outcomes [28]. While fluoroquinolone use in the community was not identified as a risk factor for this MDR infection, it was an important component of the antimicrobial stewardship and infection prevention and control bundle which was associated with decreased prevalence of this organism.

## Conclusion

Antimicrobials should be used only as necessary in treating frail elderly people, and infection control strategies such as hand and equipment hygiene are likely to be an important component of their care in hospital and in the community, to reduce the risk of future resistant UTIs. The major findings of this study illustrate the importance of all sectors of local healthcare on the transmission of MDROs, and that focusing on a single setting will not give maximal effect when implementing antimicrobial stewardship and infection prevention and control strategies to slow the transmission of these organisms.
